# Opioid dependence disorder and comorbid chronic pain: comparison of groups based on patient-attributed direction of the causal relationship between the two conditions

**DOI:** 10.1177/20494637211026339

**Published:** 2021-06-18

**Authors:** Cassie Higgins, Blair H Smith, Keith Matthews

**Affiliations:** 1 Division of Molecular and Clinical Medicine, University of Dundee, Ninewells Hospital and Medical School, Dundee, UK; 2 Division of Population Health and Genomics, University of Dundee, Ninewells Hospital and Medical School, Dundee, UK

**Keywords:** Chronic pain, pain management, opioid agonist therapy, methadone, opioid dependence, opioid use disorder, substance use disorders

## Abstract

**Background:**

Chronic pain is highly prevalent in treatment-seeking opioid-dependent patients; therefore, this comorbid presentation is an important clinical consideration for both addiction and pain specialists. The objectives of the present study were to examine whether the direction of causal attribution of opioid dependence disorder and chronic pain resulted in two distinct clinical populations, and, if so, to compare treatment received during the 5-year follow-up period.

**Methods:**

Participants comprised opioid-dependent patients with chronic pain who reported a perceived causal relationship, in either direction, between the development of these two conditions (*n* = 252). A range of health- and addiction-related instruments were used at study inception. Treatment characteristics were obtained for the follow-up period from national health registers.

**Results:**

Those reporting that pain caused opioid dependence disorder (*n* = 174; 69%) were characterised by poorer pain-related health, more illicit cannabinoid use (*p* = 0.031), more frequent illicit use of opioid analgesics (*p* = 0.025) and they were in receipt of higher doses of prescribed opioid analgesics. Those reporting that opioid dependence disorder caused pain (*n* = 78; 31%) were characterised by poorer overall physical health (*p* = 0.002), more severe psychiatric symptoms and more overall drug use (*p* = 0.001).

**Conclusion:**

Two distinct clinical populations were identified, determined by how participants perceive the causal relationship between opioid dependence disorder and chronic pain. These two populations have differing clinical profiles and treatment requirements: those reporting that pain caused opioid dependence disorder were characterised by poorer pain-related health and more illicit use of drugs with analgesic properties; and those reporting that opioid dependence disorder caused pain were characterised by more overall use of substances, multiple substance use and more intravenous substance use and poorer general health. Identifying the causal direction, where such a relationship exists, could help addiction and pain services to develop more effective, individualised treatment strategies.

## Introduction

Chronic pain (CP) is reported in up to 68% of opioid-dependent patients in receipt of opioid agonist therapy (OAT);^1^ therefore, this comorbid presentation is an important clinical consideration for both addiction and pain specialists. Patients in OAT with comorbid CP are associated with greater medical and psychiatric health burdens^2,3^ in addition to relatively severe and enduring substance dependence.^4,5^ Different disease trajectories leading to this comorbid presentation may be associated with clinical subgroups that have differing OAT and analgesic requirements and distinct profiles of medical and psychiatric morbidities that may impact on the effectiveness of standardised treatment strategies.

In the only published study to address this so far, Ilgen et al.^6^ examined the relative temporal onset of chronic, non-arthritic pain and substance use disorders (SUDs), which included alcohol, in 632 participants. They found that 56% (*n* = 351) reported that the onset of SUD preceded that of pain, 38% (*n* = 243) reported that the onset of pain preceded that of SUD and the remaining 6% (*n* = 38) reported that the onset of both conditions occurred within the same year. Ilgen’s work involved a national survey of the general population. In contrast, the present study involved a treatment-seeking sample of people diagnosed with opioid dependence disorder (ODD), and asked about their beliefs about the causal relationship in the development of CP and ODD rather than inferring this relationship based on temporal patterns. For comparison, an ICD-10 (International Classification of Diseases) diagnosis of opioid dependence (OD) generally corresponds to a *Diagnostic and Statistical Manual of Mental Disorders* (5th edition; DSM-V)^7^ diagnosis of either moderate or severe opioid use disorder. This issue is discussed in greater detail in the ‘Method’ section.

While the onset of ODD and CP may be unrelated, some patients may develop ODD as a result of analgesic opioid exposure^8,9^ or addiction-like behaviour borne out of a desire to control unmanaged pain.^10,11^ Conversely, pain may develop in patients with ODD as a result of opioid-induced hyperalgesia,^12^ which is more evident in patients with ODD than in patients with CP,^13^ or due to the higher risk of physical injury^14,15^ and the sequelae of substance use.^16^ In a comprehensive examination of this topic, Manhapra et al.^11^ suggest that patients with CP who are prescribed long-term opioid analgesia may develop an iatrogenic syndrome, referred to as complex persistent opioid dependence (CPOD). CPOD is considered as a clinical condition distinct from opioid use disorder with comorbid CP. In contrast to the clinical presentation of patients with opioid use disorder (opioid use-related social, occupational and behavioural problems), CPOD is characterised by poor pain control, declining function, psychiatric and medical instability, and aberrant behaviours.^11^ As the authors further highlight, people with CPOD require different treatment approaches to those with a primary diagnosis of opioid use disorder and are likely to feel alienated by such a diagnosis. The principal aim of the present study was to establish whether patients whose ODD led to CP presented with different clinical profiles and treatment requirements from patients whose CP led to ODD. Two hypotheses were examined:

1. Among people who had both ODD and CP, two clinically distinct groups would be identified based on the patient-attributed direction of the causal relationship between CP and ODD;
2. The characteristics associated with treatment for ODD and CP would differ between these two groups at both study inception and during the follow-up period.

## Method

### Participants and setting

Participants were drawn from the cohort of patients treated in an NHS (National Health Service) Substance Misuse Service in a Scottish Health Board area. Patients are accepted for treatment within this service if, on assessment, they are diagnosed with OD in accordance with the World Health Organization’s ICD (ICD-10, code F11.2). To avoid confusion, the term, ‘opioid dependence disorder’ (ODD), has been used throughout, since the abbreviation for ‘opioid dependence’ (OD) is commonly understood to indicate overdose. An integral component in obtaining a diagnosis of ODD is that patients must demonstrate increasing tolerance to opioids and a physical withdrawal state on cessation of opioids. On acceptance into treatment, patients commence OAT, which, at the time of the present study, comprised methadone maintenance therapy exclusively. OAT opioid prescriptions were administered by the NHS Substance Misuse Service; however, opioid analgesic prescriptions, where applicable, were administered by other NHS services, largely within the primary care setting. For comparison, there are two ICD-10 codes that correspond to a DSM-V diagnosis of OUD: (1) F11.1 (‘opioid abuse’); and (2) F11.2 (‘opioid dependence’). Generally, the former corresponds to a DSM-V diagnosis of mild OUD, and the latter corresponds to DSM-V diagnoses of moderate or severe OUD.

Participants recruited to the present study were those with CP who were in treatment in the service and who reported a perceived causal relationship, in either direction, between the development of CP and ODD. Patients who reported no causal relationship were excluded from the study since they were considered to form a relatively heterogeneous group, characterised by the absence, rather than presence, of clinical characteristics in common. Three temporal thresholds have been established to identify CP: 3, 6 and 12 months.^17^ The present study used the 12-month threshold since, in a clinical population familiar with persistent, debilitating conditions, this was considered as the best indication of truly ‘chronic’ pain, an approach that has been used in other studies.^4,18^ Those that reported having had pain for less than 12 months were excluded from the study since they were considered to form a small, relatively heterogeneous group. No participants were lost during the course of the study: as discussed below, since only anonymised data were used, participants were not required to consent to participation.

### Materials

The 9-item Brief Pain Inventory – Short Form (BPI-SF)^19^ was designed to assess the sensory and reactive dimensions of pain. The BPI-SF has demonstrated adequate validity and reliability in patients with CP^20^ and in patients receiving OAT.^21^ In the current sample, the BPI-SF evidenced excellent internal consistency (Cronbach’s α = .91). Following the administration of the BPI-SF, three additional questions were asked: (1) ‘For how long have you had your current pain problem?’; (2) ‘Do you believe that your pain problem was taken seriously by your doctor?’ and (3) ‘Do you believe that your pain problem caused your ODD, that your ODD caused your pain problem, or that they are unrelated?’.

The 60-item Maudsley Addiction Profile (MAP)^22^ was designed for research purposes in populations with drug and/or alcohol problems. This instrument was used to determine illicit use of specific substances, frequency of use (number of days used in the preceding 30 days and amount used on a typical day) and route of administration (oral, snort/sniff, smoke/case, intravenous (IV) and intramuscular). The specific illicit substances listed in the MAP are heroin, methadone, benzodiazepines, cocaine powder, crack cocaine, amphetamines and cannabis. There is also an opportunity to list other illicit substances used, including the frequency of use and the route of administration. Finally, the use and non-use of specific substances reported in the MAP was verified by performing urinalyses. It is important to note that, while the term, ‘illicit’, is used throughout this article, a proportion of patients may have been engaging in nonmedical use of substances (i.e. use of prescribed medication in a non-prescribed manner), which would apply, largely, to some patients misusing opioid analgesics and benzodiazepines that had been prescribed to them for therapeutic purposes. Scores on the MAP physical and mental health subscales range from 0 to 40, and higher scores are associated with poorer health. The MAP has demonstrated adequate validity in substance users;^22^ however, its psychometric properties have not been examined in CP samples. In the current sample, the MAP demonstrated good internal consistency (Cronbach’s α = .88).

The 28-item version of the General Health Questionnaire (GHQ-28)^23^ was designed as a screening tool to indicate any psychiatric condition. The Likert-type scoring method was used and a threshold of ⩾24 was applied (total scores range from 0 to 84). It has been shown to have the ability to accurately detect diagnoses in accordance with the Composite International Diagnostic Interview (CIDI).^24^ The GHQ-28 has demonstrated adequate validity within opioid-dependent populations;^25^ however, its psychometric properties have not been examined in CP samples. In the current sample, the GHQ-28 demonstrated good internal consistency (Cronbach’s α = .89).

The 34-item Clinical Outcomes in Routine Evaluation – Outcome Measure (CORE-OM)^26^ assesses the level of psychological global distress. Total scores range from 0 to 136, and a threshold of ⩾34 was used to indicate clinical status.^27^ The CORE-OM has demonstrated adequate validity within a healthy volunteer sample;^26^ however, its psychometric properties have not been examined in CP or opioid-dependent samples. In the current sample, the CORE-OM demonstrated acceptable internal consistency (Cronbach’s α = .80).

The 16-item Social Phobia Diagnostic Questionnaire (SPDQ)^28^ was designed as a diagnostic screening tool to identify social phobia in accordance with DSM-IV criteria. Using receiver operating characteristic analysis, the authors identified that the optimal balance between sensitivity and specificity (both >80%) was achieved using a diagnostic threshold of 7.38 (with total scores ranging from 0 to 27). The SPDQ has demonstrated adequate validity within a sample of undergraduate students;^28^ however, its psychometric properties have not been examined in CP or opioid-dependent samples. In the current sample, the SPDQ demonstrated good internal consistency (Cronbach’s α = .86).

The 10-item Treatment Perceptions Questionnaire (TPQ)^29^ was designed to measure patient satisfaction with OAT treatment. Total scores range from 0 to 40, and higher scores represent greater treatment satisfaction. The TPQ has demonstrated adequate validity within an OAT sample;^29^ however, its psychometric properties have not been examined in CP samples. In the current sample, the TPQ demonstrated acceptable internal consistency (Cronbach’s α = .78).

Demographic data were obtained from the NHS Community Health Index (CHI) dataset. These data comprised gender, age and socioeconomic status using Scottish Index of Multiple Deprivation (SIMD) quintiles (https://www.gov.scot/collections/scottish-index-of-multiple-deprivation-2020/). In accordance with SIMD recommendations, quintiles 1–2 were used to indicate relative socioeconomic deprivation and quintiles 3–5 were used to indicate relative socioeconomic affluence.

Electronic regional extracts were obtained from National Services Scotland (NSS): community-dispensed prescribing; general hospital admissions (SMR01); and psychiatric hospital admissions (SMR04).

### Procedure

The study was incepted on 1 January 2005, and the BPI-SF, substance use profiles and psychiatric assessment instruments were completed by all patients. Five-year follow-up data spanning 2005–2010 were obtained from the electronic health registers and linked at using the Community Health Index (CHI) number, a unique NHS patient identification code. Data were linked electronically by the Health Informatics Centre (HIC) Services, Dundee, a Scottish government-certified *Safe Haven*, and anonymised prior to release to the research team for analysis via a secure web link.

#### Equianalgesic computations

Morphine-equivalent doses were established using an online equianalgesic calculator based on the American Pain Society guidelines and critical review papers^30–33^ focusing on the issue of equianalgesic dosing (http://clincalc.com/opioids/). Buprenorphine was not available for conversion in the equianalgesic calculator so the Monthly Index of Medical Specialities (MIMS) conversion ratio was used, whereby, a multiplication factor of ×80 was applied to buprenorphine doses to identify morphine-equivalent doses.

#### Equianxiolytic computations

All anxiolytics prescribed in the present study were of the benzodiazepine class and 98% were diazepam; hence, all doses were converted to diazepam-equivalent doses. A wide range of conversion ratios are documented, and the ratios used in the present study (based on mode average) are detailed in the supplementary material (online Supplemental material 1).

### Statistical considerations

The Statistical Package for Social Sciences (SPSS v22) was used to undertake statistical testing. Chi-square and univariate ANOVA analyses were used to examine group differences (CP→ODD vs ODD→CP) across a number of demographic, substance use, pain-related functioning, psychiatric functioning and general functioning measures. Among demographic characteristics, chi-square was used to examine proportional differences in gender and socioeconomic status. Univariate ANOVA was used to examine differences in age. Among substance use characteristics, chi-square was used to examine proportional differences in any illicit heroin, methadone, opioid analgesic, benzodiazepine and cannabinoid use in the past, as well as across all illicit substance use. Chi-square was also used to examine proportion stabilised in OAT treatment, proportion experienced excessive hyperhidrosis in the past 4 weeks, and prior engagement in IV drug use within the past 4 weeks. Univariate ANOVA was used to assess group differences in mean number of days used in the past 30 days for each substance and mean injecting risk score. Among pain characteristics, chi-square was used to examine proportional differences in multiple sites of pain, pain interference, receipt of prescribed opioid analgesics and the perception that pain problems were taken seriously by the treating physician. Univariate ANOVA was used to assess group differences in mean duration of pain, pain intensity and opioid analgesic dose. Among psychiatric assessment characteristics, chi-square was used to examine proportional differences in meeting clinical thresholds on specific assessment instruments (GHQ-28, CORE-OM and SPDQ). Univariate ANOVA was used to assess group differences in mean scores on the GHQ-28 subscales (social dysfunction, severe depression, somatic symptoms and anxiety/insomnia) and the CORE-OM subscales (subjective wellbeing, problems/symptoms, life functioning and risk/harm). Among medical and psychiatric treatment characteristics, chi-square was used to examine proportional differences in receipt of prescribed medication and inpatient treatment. Univariate ANOVA was used to assess group differences in mean number of hospital admissions.

Since multiple comparisons can result in Type I errors (i.e. false positive results), the familywise error rate was controlled, using a manual Bonferroni’s correction. The familywise error rate (set at the conventional 0.05) was divided by the number of tests associated with one single hypothesis to establish the critical value (α) for individual tests. Where the critical value was adjusted, this was recorded in the text or table footnotes. This relates specifically to assessments of patients meeting clinical thresholds on the GHQ-28 and the CORE-OM, since it could be argued that they measure similar constructs. Clinical thresholds associated with the total scores on these instruments provide an indication of psychiatric diagnostic status. They were considered to be a family of tests and, therefore, the alpha was adjusted accordingly (*p* ⩽ 0.025).

### Ethical approval

Ethical approval was not required for the present study, since all data were anonymised and accessed via a national *Safe Haven*; however, a favourable ethical opinion was obtained from the East of Scotland Research Ethics Committee (EoSREC).

## Results

Of the 615 patients in the NHS Substance Misuse Service, 221 (36%) were excluded due to having no pain, 54 (9%) were excluded due to having pain of less than 12 months duration and 88 (14%) were excluded due to reporting no perceived causal relationship, in either direction, between the onset of CP and ODD. The remaining 252 patients (41% of the entire treatment population) comprised the present study cohort. Just over two-thirds of the study cohort (69%, *n* = 174) reported the belief that CP had caused ODD (the *CP→ODD group*) while the remainder (31%, *n* = 78) reported the belief that ODD had caused CP (the *ODD→CP group*).

### Group differences in sociodemographic characteristics and patient-reported illicit substance use at study inception

Table 1 shows that the CP→ODD group was significantly older than the ODD→CP group. While statistically significant, it equates to a mean age difference of only 2 years. A higher proportion of the ODD→CP group reported any illicit substance use in the 30 days prior to study inception. When broken down by substance category, it was found that, across the entire sample, a higher proportion of the ODD→CP group engaged in illicit use of methadone, opioid analgesics and cannabinoids in the prior 30 days. Of those who reported illicit methadone and illicit heroin use, individuals in the ODD→CP group endorsed significantly more days of use than those in the CP→ODD group. Conversely, of those who reported illicit use of opioid analgesics, individuals in the CP→ODD group endorsed significantly more days of use than those in the ODD→CP group. It is important to note that these findings pertain to self-reported illicit substance use, which does not include medication taken as prescribed.

**Table 1.** Group differences in sociodemographic characteristics and patient-reported illicit substance use at study inception.

CP→ODD			ODD→CP		
Sociodemographic characteristics	*n*	%	*n*	%	*p* value (*ω*)
Gender					0.591 (0.034)
Male	111	70	47	64	
Female	48	30	27	36	
Socioeconomic status^a^					0.260 (0.071)
Socioeconomically deprived	154	91	65	87	
Socioeconomically affluent	15	9	10	13	
	$\bar{x}$	σ	$\bar{x}$	σ	*p* value ( $\eta_{p}^{2}$ )
Mean age (years)	35	8	33	6	**0.018** (0.024)
*Any illicit use of substances in the past 30* *days*	*n*	%	*n*	%	*p* value (*ω*)
Any substance	133	89	67	100	**0.001** (0.195)
Heroin	60	40	31	46	0.249 (0.056)
Methadone	45	30	29	43	**0.041** (0.129)
Opioid analgesics	18	12	16	24	**0.022** (0.154)
Benzodiazepines	49	33	24	36	0.380 (0.031)
Cannabinoids	110	74	58	87	**0.031** (0.137)
*Mean number of days of use in the past 30* *days*	$\bar{x}$	σ	$\bar{x}$	σ	*p* value ( $\eta_{p}^{2}$ )
Heroin	6.63	9	11.06	10	**0.033** (0.050)
Methadone	7.96	10	12.97	10	**0.039** (0.058)
Opioid analgesics	8.56	11	1.94	2	**0.025** (0.147)
Benzodiazepines	9.41	11	8.79	10	0.820 (0.001)
Cannabinoids	22.05	11	22.62	11	0.751 (0.001)

CP: chronic pain; ODD: opioid dependence disorder.

a Calculated using the Scottish Index of Multiple Deprivation (SIMD), whereby quintiles 1–2 represent relative deprivation and quintiles 3–5 represent relative affluence. The bold values are the p-values that meet statistical significance.

### Group differences in the characteristics of ODD and CP at study inception

Table 2 shows that the CP→ODD group was associated with a longer pain duration and higher mean pain intensity at study inception. A higher proportion of the ODD→CP group had failed to stabilise in OAT treatment and were using drugs intravenously (IV), and this group was associated with a higher health risk as a consequence of IV drug use.

**Table 2.** Characteristics of opioid dependence disorder and chronic pain and at study inception.

CP→ODD			ODD→CP		
*Characteristics of opioid dependence disorder*					
	*n*	%	*n*	%	*p* value (*ω*)
Stabilised in OAT treatment^a^	42	25	6	8	**0.003** (0.194)
Excessive hyperhidrosis	129	77	57	75	0.702 (0.025)
Intravenous drug use within past 4 weeks	18	10	16	21	**0.030** (0.137)
	$\bar{x}$	σ	$\bar{x}$	σ	*p* value ( $\eta_{p}^{2}$ )
Mean score for injecting risk (0–48 scale)^b^	1.7	4.0	8.8	12.1	**0.042** (0.135)
*Characteristics of chronic pain*					
	*n*	%	*n*	%	*p* value (*ω*)
Pain at multiple sites	38	22	18	23	0.827 (0.014)
Pain interference: daily activities	125	76	58	77	0.790 (0.017)
Pain interference: sleep	136	78	64	83	0.368 (0.057)
	$\bar{x}$	σ	$\bar{x}$	σ	*p* value ( $\eta_{p}^{2}$ )
Mean pain duration (months)	97	93	57	58	**0.001** (0.048)
Mean pain intensity (0–100 scale)^b^	64	21	58	21	**0.040** (0.017)

CP: chronic pain; ODD: opioid dependence disorder; OAT: opioid agonist therapy.

a Stabilisation was indicated where individuals had been on a consistent daily dose and dosing schedule for at least 3 months.

b Higher scores indicate greater symptom severity. The bold values are the p-values that meet statistical significance.

### Group differences during the 5-year follow-up period in medical and psychiatric morbidity, OAT and analgesic treatment

At study inception, the ODD→CP group was associated with a higher mean score on the Physical Health subscale of the MAP (*M* = 19.39, *SD* = 7.88) compared with the CP→ODD group (*M* = 16.08, *SD* = 7.49) (*F*(1,247) = 10.034; *p* = 0.002; $\eta_{p}^{2}$ = 0.039), indicating significantly poorer physical health in this group. Table 3 shows group differences in psychiatric morbidity at study inception using standardised instruments. Assessments using both the GHQ-28 and the CORE-OM, were considered to be a family of tests and, therefore, the alpha was adjusted accordingly (*p* ⩽ 0.025).

**Table 3.** Group differences in psychiatric morbidity at study inception.

CP→ODD			ODD→CP		
	*N*	%	*n*	%	*p* value (*ω*)
Clinical threshold on GHQ-28	80	57	49	73	**0.018** (0.154)
Clinical threshold on CORE-OM	115	69	63	83	**0.017** (0.143)
Social phobia threshold (SPDQ)	56	38	42	63	**0.001** (0.231)
	$\bar{x}$	σ	$\bar{x}$	σ	*p* value ( $\eta_{p}^{2}$ )
*GHQ-28 subscales*					
Social dysfunction (0–21 scale)	7.99	3.12	9.09	3.13	**0.012** (0.027)
Severe depression (0–21 scale)	4.73	4.70	5.42	4.93	0.298 (0.005)
Somatic symptoms (0–21 scale)	7.77	3.83	8.86	3.91	**0.043** (0.017)
Anxiety/insomnia (0–21 scale)	8.45	4.99	9.84	4.73	**0.042** (0.017)
*CORE-OM subscales*					
Subjective wellbeing (0–16 scale)^a^	7.27	4.41	8.04	5.59	0.211 (0.006)
Problems/symptoms (0–48 scale)	24.0	11.4	30.0	25.2	**0.011** (0.026)
Life functioning (0–48 scale)^a^	17.9	10.8	21.5	13.1	**0.025** (0.021)
Risk/harm (0–24 scale)	2.14	3.15	3.09	3.40	**0.035** (0.018)

CP: chronic pain; ODD: opioid dependence disorder; GHQ: general health questionnaire; CORE-OM: clinical outcomes in routine evaluation-outcome measure; SPDQ: social phobia diagnostic questionnaire.

Note: The assessment of clinical thresholds using the GHQ-28 and CORE-OM were considered to be a family of test, since they assess similar domains. In consequence, the critical value for these assessments was adjusted to *p* ⩽ 0.025. The critical value for all other assessments remained at *p* ⩽ 0.05.

a These subscales are problem scored; therefore, higher scores are associated with greater symptom severity. The bold values are the p-values that meet statistical significance.

Table 3 shows that a higher proportion of the ODD→CP group was considered to be associated with a clinical psychiatric condition (i.e. scoring above the cut-off on the GHQ-28 and CORE-OM), and there was a higher prevalence of social phobia in this group. Furthermore, this group was associated with significantly higher subscale scores, indicating greater symptom severity, on three of the four GHQ-28 subscales (Social Dysfunction, Somatic Symptoms and Anxiety/Insomnia) and three of the four CORE-OM subscales (Problems/Symptoms, Life Functioning and Risk/Harm). Table 4 shows the proportion of each group that was prescribed medication or admitted to inpatient facilities for the treatment of medical or psychiatric morbidity during the 5-year follow-up period.

**Table 4.** Group differences during the 5-year follow-up period in prescribed medication and inpatient admissions for the treatment of medical and psychiatric morbidity, characteristics of prescribed OAT and analgesic medication, and patient perceptions of treatment satisfaction.

CP→ODD			ODD→CP		
Treatment for medical morbidity	*n*	%	*n*	σ	*p* value (*ω*)
Medication for medical morbidity	162	93	74	95	0.595 (0.034)
Medication excluding analgesia	155	89	72	92	0.428 (0.050)
Admission to general hospitals	81	47	31	40	0.315 (0.063)
	$\bar{x}$	σ	$\bar{x}$	σ	*p* value ( $\eta_{p}^{2}$ )
Mean number of admissions	2.6	2.2	4.6	4.7	**0.003** (0.078)
Mean duration of stay (nights)	15	21	45	79	**0.002** (0.083)
Treatment for psychiatric morbidity	*n*	%	*n*	σ	*p* value (*ω*)
Medication for psychiatric morbidity	155	89	69	89	0.885 (0.009)
Medication for anxiety disorders	115	66	55	71	0.489 (0.044)
Medication for depressive disorders	128	74	56	72	0.770 (0.018)
Medication for psychotic disorders	32	18	9	12	0.885 (0.009)
Admission to psychiatric hospitals	34	20	7	9	**0.036** (0.132)
	$\bar{x}$	σ	$\bar{x}$	σ	*p* value ( $\eta_{p}^{2}$ )
Mean number of admissions	2.12	1.49	2.43	1.72	0.627 (0.006)
Mean duration of stay (nights)	23.1	22.1	33.8	49.0	**0.002** (0.083)
Medication prescribed by Substance Misuse Service	*n*	%	*n*	σ	*p* value (*ω*)
Receipt of benzodiazepines	86	49	34	44	0.391 (0.054)
	$\bar{x}$	σ	$\bar{x}$	σ	*p* value ( $\eta_{p}^{2}$ )
Mean ME OAT methadone dose (mg/day)	104	54	108	54	0.553 (0.001)
Mean DE benzodiazepine dose (mg/day)	34	28	31	26	0.556 (0.003)
*Patient satisfaction with treatment at Substance Misuse Service*	$\bar{x}$	σ	$\bar{x}$	σ	*p* value ( $\eta_{p}^{2}$ )
OAT treatment satisfaction score (0–40 scale)^a^	23	6	21	6	**0.019** (0.023)
*Medication prescribed for analgesia*	*n*	%	*n*	σ	*p* value (*ω*)
Receipt of opioid analgesics	19	11	11	14	0.471 (0.045)
	$\bar{x}$	σ	$\bar{x}$	σ	*p* value ( $\eta_{p}^{2}$ )
Mean ME opioid analgesic dose (mg/day)^b^	64	61	25	15	**0.049** (0.132)
*Patient satisfaction with physician attitude towards pain problem*	*n*	%	*n*	σ	*p* value (*ω*)
Pain problem perceived to have been taken seriously by physician	89	63	30	56	0.332 (0.069)

CP: chronic pain; ODD: opioid dependence disorder; ME: morphine-equivalent; DE: diazepam-equivalent; OAT: opioid agonist therapy.

a Higher scores indicate greater treatment satisfaction.

b Including methadone where it was prescribed for analgesic purposes. The bold values are the p-values that meet statistical significance.

Table 4 shows that most participants in each group had been in receipt of prescribed medication for the treatment of medical morbidity at some point during the follow-up period, even after exclusion of all classes of analgesic medication. The ODD→CP group was associated with a higher number of general hospital admissions than the CP→ODD group and spent more nights in hospital. There were no group differences concerning receipt of prescribed medication for the treatment of psychiatric morbidities with the majority of both groups having been in receipt of medication for the treatment of anxiety and depressive disorders. A higher proportion of the CP→ODD group had been admitted to psychiatric hospitals during the follow-up period. Conversely, of those that were admitted, the ODD→CP group was associated with a higher mean number of nights’ stay in hospital during this period. Table 4 further shows that the CP→ODD group was in receipt of a higher morphine-equivalent opioid analgesic daily dose for the treatment of pain than the ODD→CP group. Despite this finding, there was no group difference concerning satisfaction with treatment for pain problems. There were no group differences concerning methadone dose, receipt of benzodiazepines or diazepam-equivalent daily benzodiazepine dose. The ODD→CP group was, however, less satisfied with OAT treatment.

## Discussion

Among people with ODD and comorbid CP, we identified two distinct clinical subgroups, determined by the patient-attributed causal relationship between their ODD and their CP. Our findings suggest that these two groups present as clinically distinct treatment populations. A higher proportion of the ODD→CP group reported IV drug use and illicit use of methadone, opioid analgesics and cannabinoids, and more frequent illicit use of heroin and methadone was reported in this group. More frequent illicit use of opioid analgesics was reported in the CP→ODD group. While the CP→ODD group was associated with poorer pain-related health, the ODD→CP group was associated with poorer overall physical health and more severe psychiatric symptoms, particularly general functioning and anxiety-related disorders. Furthermore, we found that the CP→ODD group was in receipt of a higher mean morphine-equivalent daily dose of opioid analgesics for the treatment of pain than the ODD→CP group but there was no difference concerning satisfaction with treatment for pain problems, with around 60% of both groups reporting satisfaction with treatment. There were no group differences concerning methadone dose, receipt of benzodiazepines or mean diazepam-equivalent daily dose; however, the ODD→CP group was less satisfied with OAT treatment.

Patients in the CP→ODD group reported poorer pain-related health and more frequent illicit use of opioid analgesics. Since analgesic effectiveness is associated with more frequent dosing than is obtained in OAT, more frequent illicit use of opioid analgesics in this group may indicate efforts to control unmanaged pain. Indeed, membership of this group was significantly associated with a longer mean duration of pain and a higher mean intensity of pain. This finding is consistent with the characteristics of CPOD described by Manhapra et al.,^11^ since the CP→ODD group appeared to be focussed primarily on addressing their pain problems. Indeed, the CP→ODD group, or a substantial proportion of it, may be experiencing CPOD. A higher proportion of the ODD→CP group reported illicit drug use, use of multiple substances, more IV drug use and, generally, more frequent use of illicit substances. This is similarly consistent with the characteristics of substance misuse discussed by Manhapra et al.:^11^ this complex profile may indicate that many patients in this group continued to struggle with illicit substance use. Indeed, a smaller proportion of this group was reported to be stabilised in OAT, and a higher proportion was associated with risk of harm due to drug use. Furthermore, the ODD→CP group was in receipt of a lower analgesic dose (but were equally satisfied with analgesic treatment) and on a comparable methadone dose (but were less satisfied than the CP→ODD group with OAT treatment). As also suggested by Manhapra et al.,^11^ it appears that this group perceives ODD to be their primary problem and, in consequence, a proportion may perceive that further OAT treatment, rather than analgesic treatment, would help to attenuate their problems.

Patients in the ODD→CP group were associated with more psychiatric morbidity, significantly higher psychiatric subscale scores and significantly more nights spent in psychiatric inpatient facilities. This finding is contrary to that of Ilgen et al.,^6^ who found no group differences concerning lifetime mood and anxiety disorders. Higher rates of psychiatric problems are commonly a function of affective distress states associated with opioid withdrawal,^34,35^ and, in consequence, the findings of their study may have been ‘diluted’ by the inclusion of patients with other drug and alcohol disorders. Patients in the ODD→CP group were also associated with poorer physical health, a higher number of admissions to general hospitals and a higher mean number of nights spent in inpatient facilities. These findings are contrary to the work of Manhapra et al.,^11^ who suggest that people with a primary pain problem (i.e. patients with CPOD), rather than a primary opioid use problem, are likely to experience medical and psychiatric instability. They did not, however, characterise opioid-dependent people who subsequently develop pain. It may be that opioid-dependent patients with comorbid CP present with relatively more complex medical and psychiatric complications than those without pain – indeed our previous work has shown this in relation to psychiatric disorders.^18^ Future research studies might consider including a third, comparator group – those with opioid use problems and no pain problems.

This is the first study to examine both the patient-attributed causal direction of ODD and CP and its association with health and clinical factors. Ilgen et al.^6^ examined the relative temporal onset of pain and SUDs. There is an obvious link between exposure to opioids and ODD, and the inclusion in Ilgen’s cohort of patients who had had any SUD (rather than focusing specifically on opioids) may have resulted in a dilution of any real effect of opioid use disorder on the direction of attribution. Furthermore, in contrast to Ilgen’s approach, the present study adopted a patient-centred approach, in a methadone-maintained sample, asking about patients’ beliefs about the causal relationship in the development of CP and ODD rather than inferring this relationship based on temporal patterns.

All patients in both of these groups were maintained on methadone, and this may not be the best available treatment for these groups. Certainly, the CP→ODD group appears strikingly similar to the CPOD patients described by Manhapra et al.,^11^ with poorer pain-related health and more frequent use of substances with analgesic properties. Therefore, the principal requirement for this group of patients is the delivery of effective analgesia while minimising harm. This might involve transitioning to non-opioid analgesics or methadone dose titration;^9^ however, many patients struggle with this approach, developing greater pain severity and pain-related functional impairment.^11^ In this case, an alternative approach might involve opioid substitution with buprenorphine (a partial opioid agonist),^9^ to minimise the harms associated with methadone. However, it should be noted that there is evidence to suggest that buprenorphine is relatively ineffective at reducing pain intensity in patients with comorbid opioid use disorder.^36^ Given that pain appears to be the driver for aberrant behaviours in this group, these patients may find non-pharmacological interventions – such as physical therapy and training in self-management strategies – to be more effective in managing their pain while reducing the risk of exacerbating their OD. It is difficult to comment in detail on the appropriateness of methadone treatment for the ODD→CP group, and future studies should consider comparing functional outcomes in this group with those of ODD patients with no pain. It is clear, however, that patients in this group could benefit from inter-agency approaches involving general and addiction psychiatric services alongside specialist pain services. Our method of directly asking patients about the causal relationship between CP and ODD, while serving its intended purpose in this study, is not recommended for use in clinical practice. Accurately responding to this question demands an understanding of the complexities in the relationship between CP and ODD, complete candour and faultless recall – unrealistic demands to place upon patients. Instead, there may be a valuable role for general practitioners, as the coordinators of medical care, in carefully monitoring disease trajectories as symptoms develop and ensuring that the most appropriate care services are provided.

This is a novel study that examines clinical characteristics in OAT patients with CP as a function of the causal relationship between the development of these two conditions. The findings suggest that they present as two clinically distinct groups; however, further research is required in this area. Future studies may consider including a third, comparator group comprised of those with ODD and no pain. Furthermore, the present study reported descriptive statistics; however, the use of inferential statistics could facilitate the identification of causal links regarding CP, OD and functional outcomes. In order to direct policy and practice, however, experimental studies are required that can demonstrate the effectiveness of appropriate treatment interventions in each of these clinically distinct patient groups.

### Limitations

The reliance on patients to determine the presence and direction of any causal relationship in the development of CP and ODD could have resulted in a degree of misclassification between groups, since some may have attempted to justify drug use as attempts to bring about analgesia. The stigma associated with opioid misuse – and, more broadly, substance misuse – is culturally pervasive and multifaceted and can, ultimately, result in non-engagement or disengagement from treatment.^37^ In addition, patients with severe CP may find it more difficult to access care due to mobility issues or other functional impairments, and this could result in further sample bias. Finally, descriptive statistical analyses are presented in this article; future studies should consider using inferential statistics to facilitate the identification of causal links regarding CP, OD and functional outcomes.

### Conclusion

Two clinically distinct treatment populations were identified based on the causal attribution of these two disorders. Those reporting that CP caused ODD were characterised by poorer pain-related health and more frequent illicit use of opioid analgesics. A higher proportion of those reporting that ODD caused CP was associated with illicit use of methadone, opioid analgesics and cannabinoids and IV drug use; this group also reported more frequent illicit use of heroin and methadone and was associated with poorer general health. In patients with both ODD and CP, establishing whether there is a causal relationship between the two disorders and, if so, identifying the direction of that relationship could help pain services to develop more effective, individualised treatment strategies. For patients reporting that ODD caused CP, this might include inter-agency approaches involving general and addiction psychiatric services alongside specialist pain services. For patients reporting that CP caused ODD, this may include careful monitoring of pain-related health and substance use and the delivery of effective analgesia while minimising harm using techniques such as dose titration, opioid substitution, transitioning to non-opioid analgesics, physical therapy and training in self-management strategies.

## Supplemental Material

sj-pdf-1-bjp-10.1177_20494637211026339 – Supplemental material for Opioid dependence disorder and comorbid chronic pain: comparison of groups based on patient-attributed direction of the causal relationship between the two conditions Click here for additional data file. Supplemental material, sj-pdf-1-bjp-10.1177_20494637211026339 for Opioid dependence disorder and comorbid chronic pain: comparison of groups based on patient-attributed direction of the causal relationship between the two conditions by Cassie Higgins, Blair H Smith and Keith Matthews in British Journal of Pain
