# Supplementary material for: Opioid dependence disorder and comorbid chronic pain: comparison of groups based on patient-attributed direction of the causal relationship between the two conditions
Source: Br J Pain. 2021 Jun 18;16(2):149–60. doi: 10.1177/20494637211026339 (PMC8998531; doi:10.1177/20494637211026339)
Supplement: sj-pdf-1-bjp-10.1177_20494637211026339 – Supplemental material for Opioid dependence disorder and comorbid chronic pain: comparison of groups based on patient-attributed direction of the causal relationship between the two conditions [file sj-pdf-1-bjp-10.1177_20494637211026339.docx]

***Table S1:*** *Common diazepam-equivalent conversion ratios and selection of the ratios for use in the present study*

|  | **Diazepam** | **Chlordiazepoxide** | **Lorazepam** | **Oxazepam** |
| --- | --- | --- | --- | --- |
| BNF (2012) | 5mg | 15mg | 0.5-1mg | 15mg |
| Taylor *et al.* (2012) | 5mg | 12.5mg | 0.5mg | 15mg |
| Bazire (2012)^a^ | 5mg | 15mg  (10-25mg) | 0.5-1mg at 4mg/d  (2mg at 5mg/d) | 15mg  (10-40mg) |
| DoH (2007) | 5mg | 15mg | 0.5mg | 15mg |
| Ashton (2002)^b^ | 5mg | 12.5mg | 0.5mg | 10mg |
| ClinCalc (2016) | 5mg | 25mg  (6-25mg) | 1mg  (0.25-2mg) | 10mg  (2.5-20mg) |
| ***Present study*** | ***5mg*** | ***15mg*** | ***0.5mg*** | ***15mg*** |
